# Altered Vision-Related Resting-State Activity in Pituitary Adenoma Patients with Visual Damage

**DOI:** 10.1371/journal.pone.0160119

**Published:** 2016-08-11

**Authors:** Haiyan Qian, Xingchao Wang, Zhongyan Wang, Zhenmin Wang, Pinan Liu

**Affiliations:** 1 Department of Neurosurgery, Beijing Tiantan Hospital affiliated to Capital Medical University, Beijing, China; 2 Department of Radiology, Beijing Tiantan Hospital affiliated to Capital Medical University, Beijing, China; 3 Beijing Neurosurgery Institute, Capital Medical University affiliated to Capital Medical University, Beijing, China; University of Montreal, CANADA

## Abstract

**Objective:**

To investigate changes of vision-related resting-state activity in pituitary adenoma (PA) patients with visual damage through comparison to healthy controls (HCs).

**Methods:**

25 PA patients with visual damage and 25 age- and sex-matched corrected-to-normal-vision HCs underwent a complete neuro-ophthalmologic evaluation, including automated perimetry, fundus examinations, and a magnetic resonance imaging (MRI) protocol, including structural and resting-state fMRI (RS-fMRI) sequences. The regional homogeneity (ReHo) of the vision-related cortex and the functional connectivity (FC) of 6 seeds within the visual cortex (the primary visual cortex (V1), the secondary visual cortex (V2), and the middle temporal visual cortex (MT+)) were evaluated. Two-sample t-tests were conducted to identify the differences between the two groups.

**Results:**

Compared with the HCs, the PA group exhibited reduced ReHo in the bilateral V1, V2, V3, fusiform, MT+, BA37, thalamus, postcentral gyrus and left precentral gyrus and increased ReHo in the precuneus, prefrontal cortex, posterior cingulate cortex (PCC), anterior cingulate cortex (ACC), insula, supramarginal gyrus (SMG), and putamen. Compared with the HCs, V1, V2, and MT+ in the PAs exhibited decreased FC with the V1, V2, MT+, fusiform, BA37, and increased FC primarily in the bilateral temporal lobe (especially BA20,21,22), prefrontal cortex, PCC, insular, angular gyrus, ACC, pre-SMA, SMG, hippocampal formation, caudate and putamen. It is worth mentioning that compared with HCs, V1 in PAs exhibited decreased or similar FC with the thalamus, whereas V2 and MT+ exhibited increased FCs with the thalamus, especially pulvinar.

**Conclusions:**

In our study, we identified significant neural reorganization in the vision-related cortex of PA patients with visual damage compared with HCs. Most subareas within the visual cortex exhibited remarkable neural dysfunction. Some subareas, including the MT+ and V2, exhibited enhanced FC with the thalamic pulvinar, which indicates an important role in the compensatory mechanism following visual impairment. In addition, neural dysfunction within the visual cortex was associated with neural activity alternation in the higher-order cognitive cortex, especially subareas in default mode network (DMN) and salience network (SN).

## Introduction

The visual cortex is composed of more than 50 subareas, and each area has specific functions and connectivities [1]. The main stream of visual information from the retina is relayed to V1 (Brodmann area 17) via the lateral geniculate nucleus (LGN) through V2 (Brodmann area 18), to higher-order visual cortex[2–7]. The synaptic connections of retinofugal neurons on area MT+ relay cells in the pulvinar and LGN have been identified, which suggests an alternative pathway that bypasses V1 directly to MT+/V5 [8, 9]. This bypass advances MT+ as a potential substitute following a lesion at early age in V1, a notion that has been confirmed in previous studies [8, 10, 11]. Previous studies regarding visual cortex plasticity predominately recruited individuals with cortical lesion or blindness at early age. It remains unclear whether MT+ still, or if not, other subarea in visual cortex, play the role for compensation for partially vision-deprivation at adulthood with intact V1.

An increasing number of studies have adopted a large-scale conjoint network perspective rather than the previously preferred individual brain area paradigm [12]. In congenitally blind individuals, the visual cortex exhibits decreased functional connectivity (FC) with the frontal motor, parietal somatosensory and temporal multi-sensory areas [13]; however, increased FC has been observed with the inferior frontal triangular areas [14]. The betweenness centrality of the SMA and hippocampus is negatively correlated with the age of onset of blindness [15]. Accumulating evidence demonstrates that the visual cortex dynamically interacts with higher cognitive areas [16–21]. To date, the studies exploring the connection between vision cortex and other higher cognitive areas mainly focused on early blind or normal-sighted people. The function connection changes between vision cortex and other higher cognitive areas associated with partially vision-deprivation at adulthood remains unclear.

Pituitary adenoma originates from the anterior lobe of the pituitary gland. When pituitary adenoma grows large enough and compresses the visual pathway, typically in the optic chiasm, optic nerve, or optic tract, patients often present with visual deficiency, including impaired visual acuity and/or visual field defects. Our study recruited pituitary adenoma patients with visual damage because vision-damage in PA patients usually occurs at adulthood due to anterior visual pathway lesion instead of visual cortex disease. The study was aimed to explore the plasticity of the visual cortex in partial visual deprived patients with intact V1 by examining the functional changes of both specific subareas within the visual cortex, especially V1, V2, and MT+, along with higher cognitive cortex beyond the visual cortex.

## Materials and Methods

### Study population

PAs were recruited from the Neurosurgery Department of Beijing Tiantan Hospital. A group of age- and sex-matched HCs with normal vision or corrected-to-normal vision were simultaneously recruited from Beijing Tiantan Hospital or local communities. Both patients and HCs were required to have no ophthalmologic diseases or other intracranial lesion that involved the visual pathway or cortex, as assessed by a neuro-ophthalmologic evaluation (see details below) and magnetic resonance imaging (MRI). PAs were selected according to the following inclusion criteria: aged 18–60 years old; corrected visual acuity below 1.0 (20/20) or visual field defect greater than 50% at least unilaterally; and tumor size not sufficiently large to distort or displace the visual cortex.

### Standard protocol approval, registration, and patient consent

This study was approved by the Institutional Review Board of Beijing Tiantan Hospital affiliated to Capital Medical University and an informed consent form was signed by all participants.

### Clinical and neuro-ophthalmologic assessment

The cognitive ability of all participants was evaluated with the Mini-Mental State Examination (MMSE), which has been widely used to clinically screen cognitive impairment prior to MRI [22]. Both patients and HCs underwent a complete neuro-ophthalmologic examination within 2 weeks of the MRI scan. The best-corrected visual acuity for distance was measured with the E chart (using the same principle as Snellen’s distant vision chart) and reported on a decimal scale. Visual field examination was performed with a standardized automated perimetry (Octopus 900 Perimetry, Switherland) were obtained. Ophthalmic fundus examination was performed with non-mydriatic retinal camera (Topcon, Japan).

### MRI scanning protocol

All functional and structural images were acquired on a 3.0 Tesla scanner (Siemens Trio, Erlangen, Germany) using a 12-channel head coil. Head movement was minimized using foam pads, and earplugs were used to attenuate acoustic noise during scanning. During the resting-state functional MRI (RS-fMRI) scan, the participants were instructed to hold still, keep their eyes closed, stay awake, and avoid thinking about anything systematic. RS-fMRI data were acquired using an echo-planar image pulse sequence (41 axial slices, slice thickness/gap = 3.5/0.7 mm, repetition time = 2500 ms, echo time = 30 ms, flip angle = 90°, and field of view (FOV) = 240 × 240 mm^2^ with an in-plane resolution of 3.75 × 3.75 mm^2^). A T1-weighted sagittal anatomical image was also obtained using a gradient echo sequence (176 slices, slice thickness/gap = 1/0 mm, inversion time = 900 ms, repetition time = 2300 ms, echo time = 3 ms, flip angle = 7°, number of excitations = 1, and FOV = 240 × 240 mm^2^ with an in-plane resolution of 0.9375× 0.9375 mm^2^).

### Data preprocessing

The resting-state fMRI data were preprocessed using SPM8 (http://www.fil.ion.ucl.ac.uk/spm) and the pipeline analysis toolbox DPARSF (http://www.restfmri.net/) [23]. To avoid transient signal changes before the longitudinal magnetization reached a steady state, the first ten volumes were discarded. The remaining images were preprocessed using a procedure that included slice timing correction, head motion correction, T1-weighted image based spatial normalization to the Montreal Neurological Institute (MNI) space, linear trend removal, and band-pass filtering (0.01–0.08 Hz). The head motion parameters of the participants were less than 3 mm in translation and less than 3 degrees in rotation. To further reduce the effects of head motion on the estimates of RS activity, we censored volumes within each participant’s fMRI time series that were associated with sudden head motion [24, 25]. For each participant, the fMRI volumes were censored if the frame-wise displacement (FD) of the head position, which was calculated as the sum of the absolute values of the derivatives of the realignment estimates, was greater than 0.5.

### Regional homogeneity (ReHo) analysis

ReHo was used to measure local synchronization of spontaneous BOLD fluctuations within a given cluster (e.g.27 nearest neighboring voxels). Kendall’s coefficient of concordance (which ranged from 0 to 1) was used as a measurement of ReHo for each voxel, which indicates the similarity between the time series of that voxel and its nearest neighboring voxels [26]. This coefficient was measured voxel-wise for each participant within a whole brain mask provided by REST [27]. To reduce nuisance sources of variation [28], individual ReHo maps were divided by the global mean value within the whole brain mask for normalization. All normalized ReHo maps were then spatially smoothed with a 6-mm full width at half-maximum (FWHM) Gaussian isotropic kernel. Two-sample t-tests based on the ReHo maps were used to analyze the differences in the vision RS activities of the HC and patient groups. The AlphaSim method, which has been implemented in REST, was used to correct for multiple comparisons. The corrected value of p < 0.05 (uncorrected p < 0.001 and minimum of 60 voxels in a cluster) was used as the threshold.

### Seed-based RS FC analysis

An FC analysis is used to evaluate correlations in activation among spatially distinct brain regions, which reflect the coherence of neural activity in different brain regions [29]. We used a seed-based RS FC analysis in our study.

#### Definition of regions of interest (ROIs)

**Fig 1:**
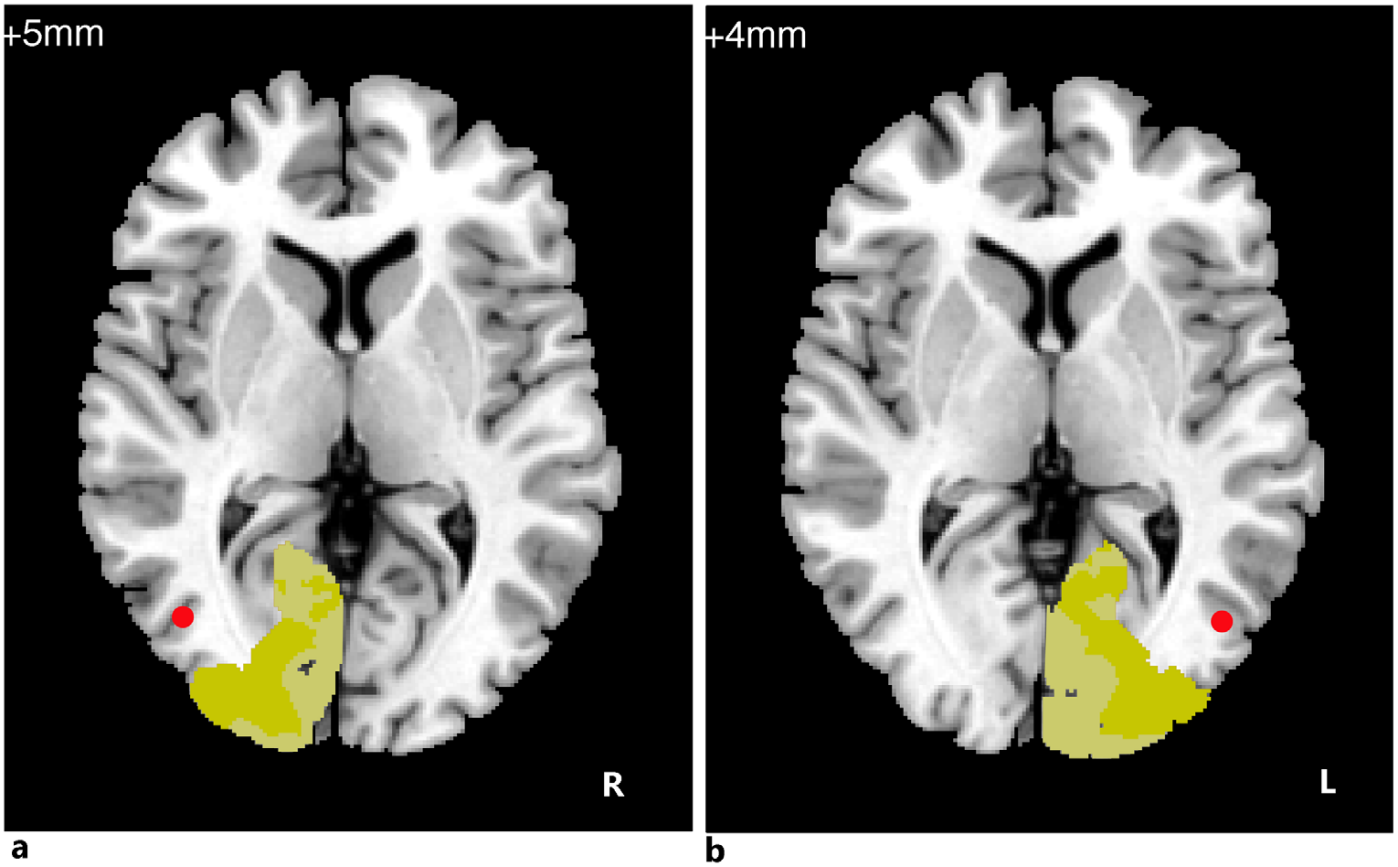
Definition of 6 seed ROIs. (a) Right MT+ (red), right BA17 (light yellow), right BA18 (dark yellow). (b) Left MT+ (red), left BA17 (light yellow), left BA18 (dark yellow).

V1 and V2 are considered to be early visual cortex. MT+, with the bypass route from the pulvinar and LGN, can also directly receive visual information. Both V1 and area MT+ develop and mature with a similar temporal profile. Because all of V1, V2 and MT+ are essential for visual information transmission from the thalamus to higher visual cortex, the bilateral V1, V2, and V5/MT+ were defined as the seed regions instead of definition based on ReHo analysis. In general, V1 refers to Brodmann area (BA) 17, whereas V2 refers to BA18. Each side of BA17 and each side of BA18 were separately chosen as ROIs. The location of human area MT+(or V5) has been correlated with the intersection of the ascending limb of the inferior temporal sulcus and the lateral occipital sulcus [30]. Because MT+ is small and hidden within the sulcus, a visual motion task is often used to help locate it [31]. However, some patients were not able to finish the visual motion task in our study because of poor vision. Therefore, we defined V5 based on data from the study by Bendy et al (right V5 coordinates: 43, -69, 5; left V5: -45, -70, 4) [32–34]. The seed ROI of V5/MT+ is defined as brain area within 6-mm radius around the coordinate. (Fig 1).

#### Functional connectivity and statistical analysis

First, noise-related variance, including six parameters of head motion correction, the average time course of the whole brain (global mean signal), the average time course within the white matter mask, and the average time course within the CSF mask, were removed from the preprocessed data by linear regression analysis. The images were then spatially smoothed with a 6-mm FWHM Gaussian kernel. We computed the FC between each seed region and each voxel within the whole brain mask. To improve data normality, the individual FC maps were transformed to z-maps using Fisher’s z-transformation. The z-values were entered into a voxel-wise two-sample t-test to determine the brain regions that exhibited significant differences in correlation between the two groups with the separate seed regions. The AlphaSim method, which has been implemented in REST, was used to correct for multiple comparisons. The corrected value of p < 0.05 (uncorrected p < 0.001 and a minimum of 60 voxels in a cluster) was used as the threshold.

## Results

Studied population. The clinical and demographic data are summarized in Table 1.

**Table 1:** Study population characteristics.

	NO.	F/M	Mean age, years (range)	Mean education, years (range)	Mean MMSE, Range
**HCs**	25	10/15	39.88 (18–60)	14.24 (9–22)	28.5
**Patients**	25	10/15	39.96 (18–60)	12.32 (9–19)	27.8

Twenty-five pituitary tumor patients with related visual damage and 25 age- and sex-matched HCs were enrolled. In total, 29 patients and 32 HCs were recruited. We excluded 4 patients and 7 controls because of head motion or the lack of sufficient data after scrubbing.

Ophthalmologic evaluation. The detailed results of the neuro-ophthalmologic evaluations are reported in Table 2. The ophthalmological examinations demonstrated that the corrected visual acuity was below 1.0 or the visual field defect was greater than 50% at least unilaterally in all patients.

**Table 2:** Ophthalmologic data of the pituitary tumor patients.

ID	Sex	Age	Vision impairment onset & side (by complaint)	Visual acuity (left)	Visual acuity (right)	Vision field defect (left) (%)	Vision field defect (right)
**1**	M	31	3 weeks (right)	1.2	0.4	25% (mainly temporal defect)	25% (mainly temporal defect)
**2**	M	60	1 month (both)	0.2	0.8	20% (superior temporal quadrant defect)	10% (superior temporal quadrant defect)
**3**	F	59	7 months (left)	Counting figures from 10 cm	1.0	unable to finish the exam	near-normal
**4**	F	42	2 years (both)	0.25	0.2	75% (only inferior nasal quadrant intact)	75% (only inferior nasal quadrant intact)
**5**	M	23	6 months (both)	0.02	0.6	40% (mainly temporal and superior nasal quadrant)	40% (mainly temporal and superior nasal quadrant)
**6**	F	59	1 year (both)	0.2	0.15	50% (mainly temporal quadrant)	25% (mainly superior temporal quadrant)
**7**	F	36	4 months (right)	0.6	No light perception	20% (mainly superior temporal quadrant)	unable to finish the exam
**8**	F	29	3 years (both)	0.8	1.2	50% (mainly temporal quadrant)	40% (mainly temporal quadrant)
**9**	F	60	5 years (both)	Hand movement from 20 cm	0.03	unable to finish the exam	90% (only spots of right inferior temporal quadrant intact)
**10**	M	38	10 months (both)	0.8	0.5	40% (mainly temporal quadrant)	40% (mainly temporal quadrant)
**11**	M	40	2 months (left)	1.0	1.0	60% (nasal and inferior temporal quadrant)	45% (mainly superior quadrant)
**12**	F	59	5 months (both)	0.6	figure counting from 20 cm	75% (temporal and superior nasal quadrant)	unable to finish the exam
**13**	M	37	1 month (left)	0.02	1.5	50% (temporal quadrant)	near-normal
**14**	M	43	1.5 years (both)	0.15	0.6	90% (tubular visual field)	50% (temporal quadrant)
**15**	M	26	7 months (both)	0.15	0.6	75% (mainly temporal and inferior nasal quadrant)	30% (mainly temporal quadrant)
**16**	M	50	4 months (right)	0.1	0.6	70% (mainly temporal and inferior nasal quadrant)	25% (inferior quadrant)
**17**	M	18	1 month (both)	0.6	0.03	75% (mainly temporal and inferior nasal quadrant)	unable to finish the exam
**18**	M	55	7 years (both)	0.5	hand movement from 10 cm	80% (only part of nasal quadrant intact)	unable to finish the exam
**19**	M	29	2 years (both)	0.1	0.6	50% (temporal heminopsia)	50% (temporal heminopsia)
**20**	M	43	5 months (left)	0.15	1.2	80% (temporal and part of nasal quadrant)	25% (superior temporal quadrant)
**21**	F	23	2 months (both)	0.3	0.05	50% (temporal heminopsia)	75% (temporal and inferior nasal quadrant)
**22**	F	23	9 days (both)	0.25	0.05	10% (temporal quadrant)	near-normal (only a reduction in sensitivity)
**23**	M	25	6 months (both)	0.4	0.4	50% (temporal heminopsia)	50% (temporal heminopsia)
**24**	F	42	1 year (both)	0.1	1	50% (temporal heminopsia)	50% (temporal heminopsia)
**25**	M	49	6 months (left)	light perception	1.2	unable to finish the exam	near-normal

### RS-fMRI analysis

#### ReHo analysis

**Fig 2:**
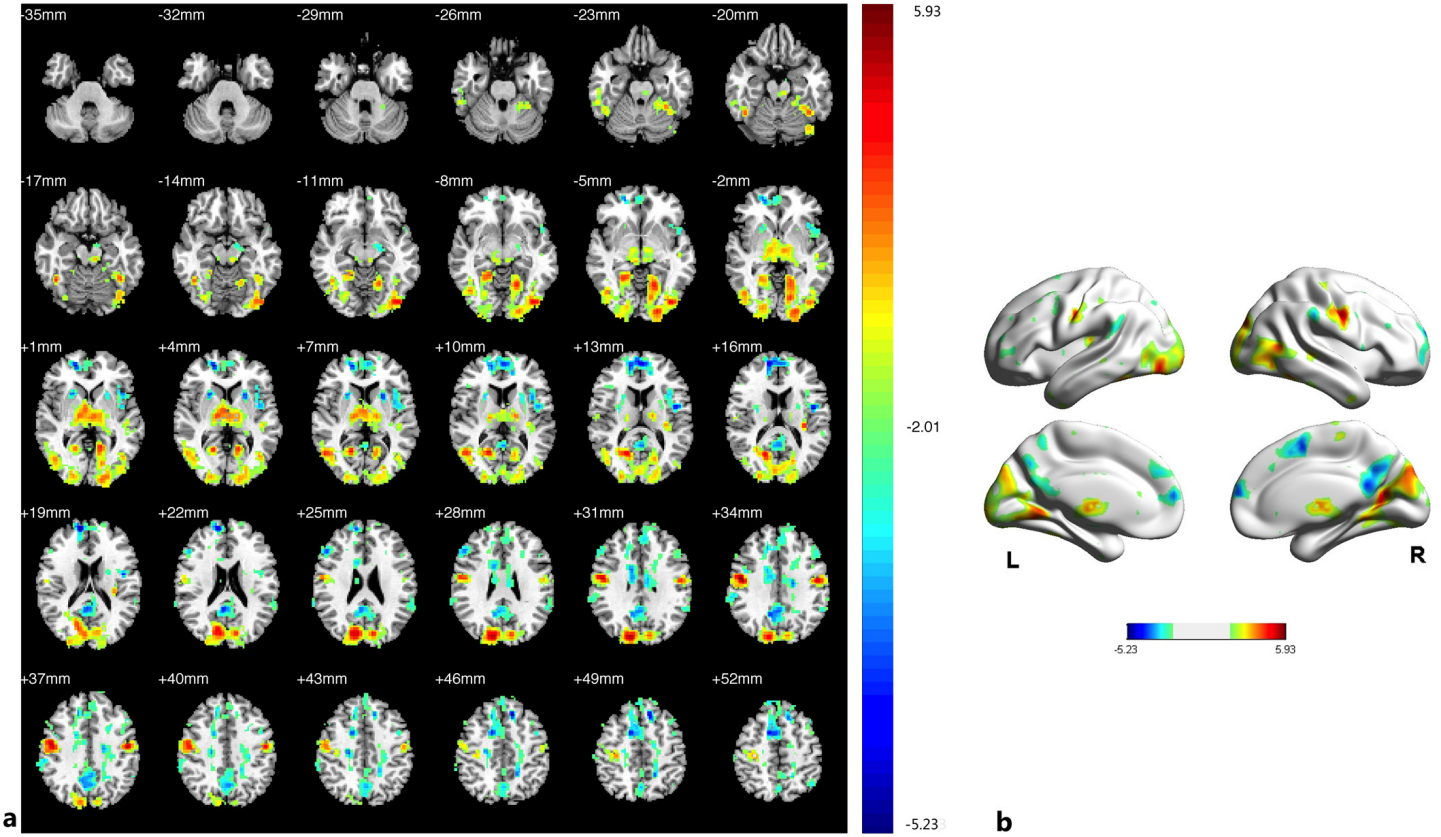
Group difference in ReHo between two groups of participants. (a) Statistical parametric map (axial view). (b) Brain areas with significant differences are shown as projected onto a 3D brain model with the BrainNet viewer. In (a), (b), the cyan-blue colors indicate increased ReHo in the PAs compared with the HCs, whereas the yellow-red colors indicate decreased ReHo. (p<0.05, AlphaSim corrected).

Compared with the HCs, the PAs exhibited a reduced ReHo in the bilateral V1, V2, V3, fusiform, MT+, BA37, thalamus, postcentral gyrus and left precentral gyrus; the patients exhibited increased ReHo in the bilateral precuneus gyrus, prefrontal cortex, ACC, PCC, insula, SMG, and putamen (Fig 2a, Table 3, See the original data in S1 File). To better visualize the differences in ReHo values between the studied groups, all clusters with significantly differential effects were also reconstructed as 3D volumes (Fig 2b).

**Table 3:** Group difference in ReHo between two groups of participants.

Brain region	Peak intensity	Peak MNI coordinate			Cluster size (voxels)
		x	y	z	
**V1(L)**	3.33592	-12	-97	3	56*
**V1(R)**	4.4178	17	-64	9	56*
**V2(L)**	4.12108	-22	-90	-5	397*
**V2(R)**	3.4079	19	-91	-3	397*
**V3(L)**	4.00769	-12	-83	29	352*
**V3(R)**	5.1207	15	-83	25	352*
**MT+BA37(L)**	3.30991	-45	-70	4	90*
**MT+BA37(R)**	2.90961	43	-69	5	90*
**Fusiform(L)**	4.21677	-40	-48	-20	137
**Fusiform(R)**	3.87639	43	-48	-21	126
**Thalamus(L)**	3.95477	-3	-14	3	163
**Thalamus(R)**	4.15292	13	-13	3	132
**Post+precentral G(L)**	4.01204	-55	-10	33	142
**postcentral G(R)**	5.49065	47	-10	32	103
**Precuneus(L)**	-3.52014	-3	-62	38	316*
**Precuneus(R)**	-3.82791	8	-56	34	316*
**Prefrontal(L)**	-3.9789	0	59	12	241*
**Prefrontal(R)**	-4.75761	11	59	18	241*
**PCC(L)**	-3.43992	-2	-47	20	117*
**PCC(R)**	-3.92524	6	-47	23	117*
**ACC(L)**	-3.39646	-13	45	10	51*
**ACC(R)**	-3.30964	13	45	13	51*
**Insula(L)**	-3.63426	-43	17	0	63
**Supramarginal(L)**	-2.91934	-57	-50	32	45
**Supramarginal(R)**	-3.00721	63	-32	35	80
**Putamen(L)**	-3.96993	-22	14	3	34
**Putamen(R)**	-3.48148	22	14	2	47

Note: MT+ is too small to distinguish it from BA37, so we took MT+ and BA37 as a whole to calculate the overall cluster size. For some BA areas whose cluster size REST Slice Viewer didn’t report in each cerebrum separately, overall cluster size of both cerebri was reported, which was marked by *. (Tables 3–9)

#### FC analysis

The temporal synchrony during rest between six seed ROIs (bilateral V1, V2, and V5/MT+) and other brain regions was assessed using a correlation analysis. A voxel-wise two-sample t-test was subsequently used to determine the brain regions that exhibited significant differences between the PAs and HCs in the FC of each side of V1, V2, and V5/MT+ with other brain regions.

**Fig 3:**
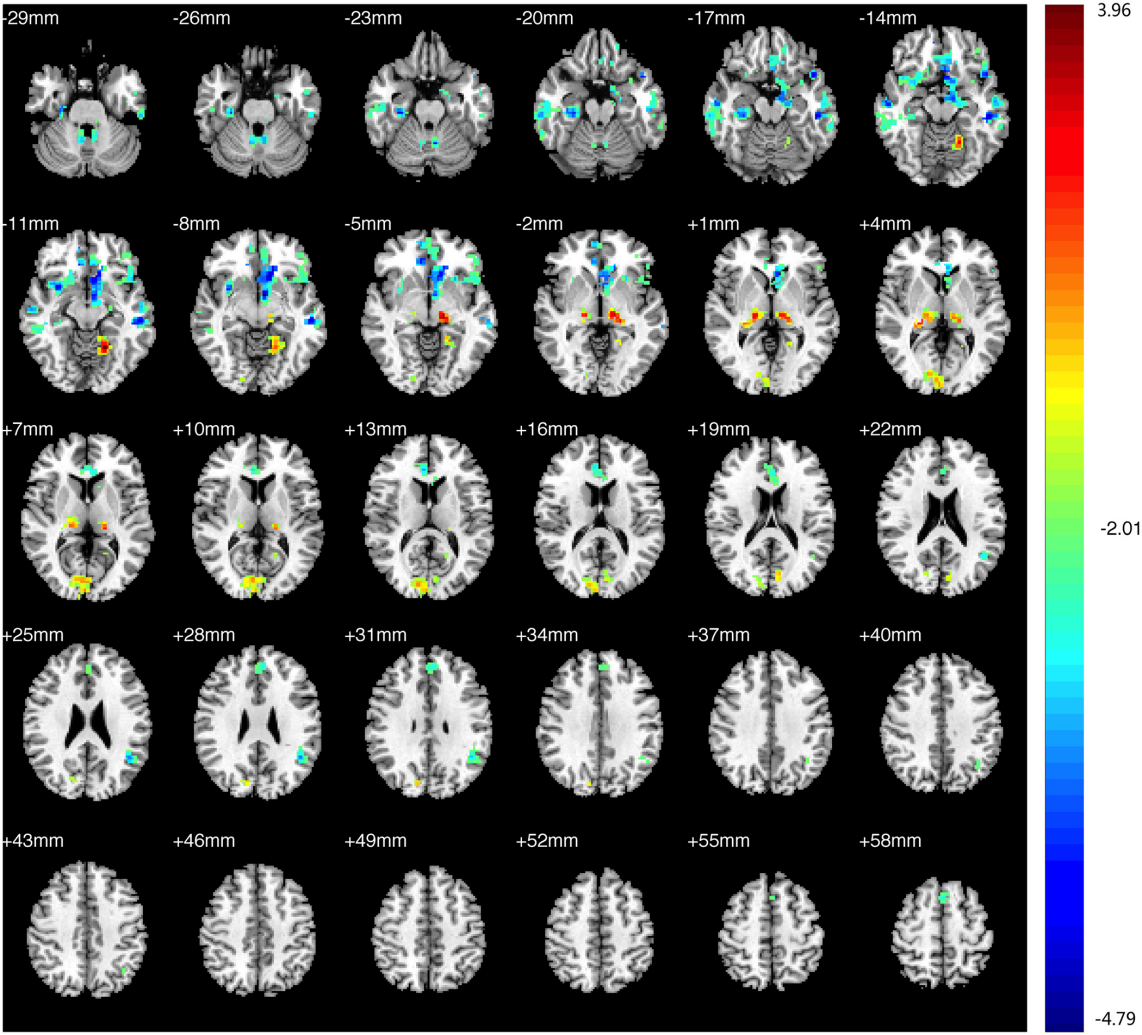
Brain areas exhibited significantly different FCs with the left V1 in PAs compared with HCs. Statistical parametric map (axial view). The cyan-blue colors indicate increased FC with the left V1 in the PAs versus the HCs, whereas the yellow-red colors indicate decreased FC in the same comparison. (p<0.05, AlphaSim corrected).

**Fig 4:**
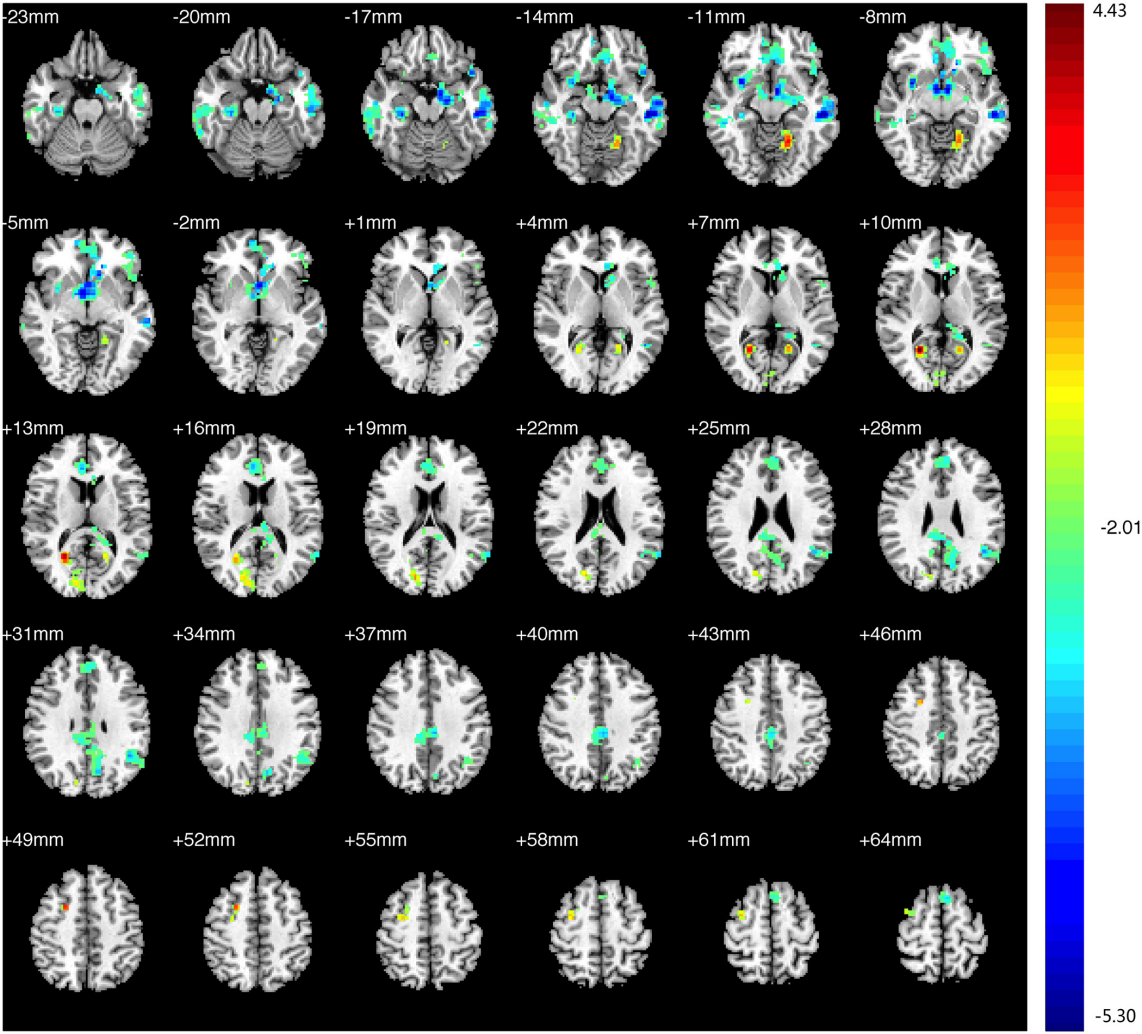
Brain areas exhibited significantly different FCs with the right V1 in PAs compared with HCs. Statistical parametric map (axial view). The cyan-blue colors indicate increased FC with the right V1 in the PAs versus the HCs, whereas the yellow-red colors indicate decreased FC. (p<0.05, AlphaSim corrected).

**Fig 5:**
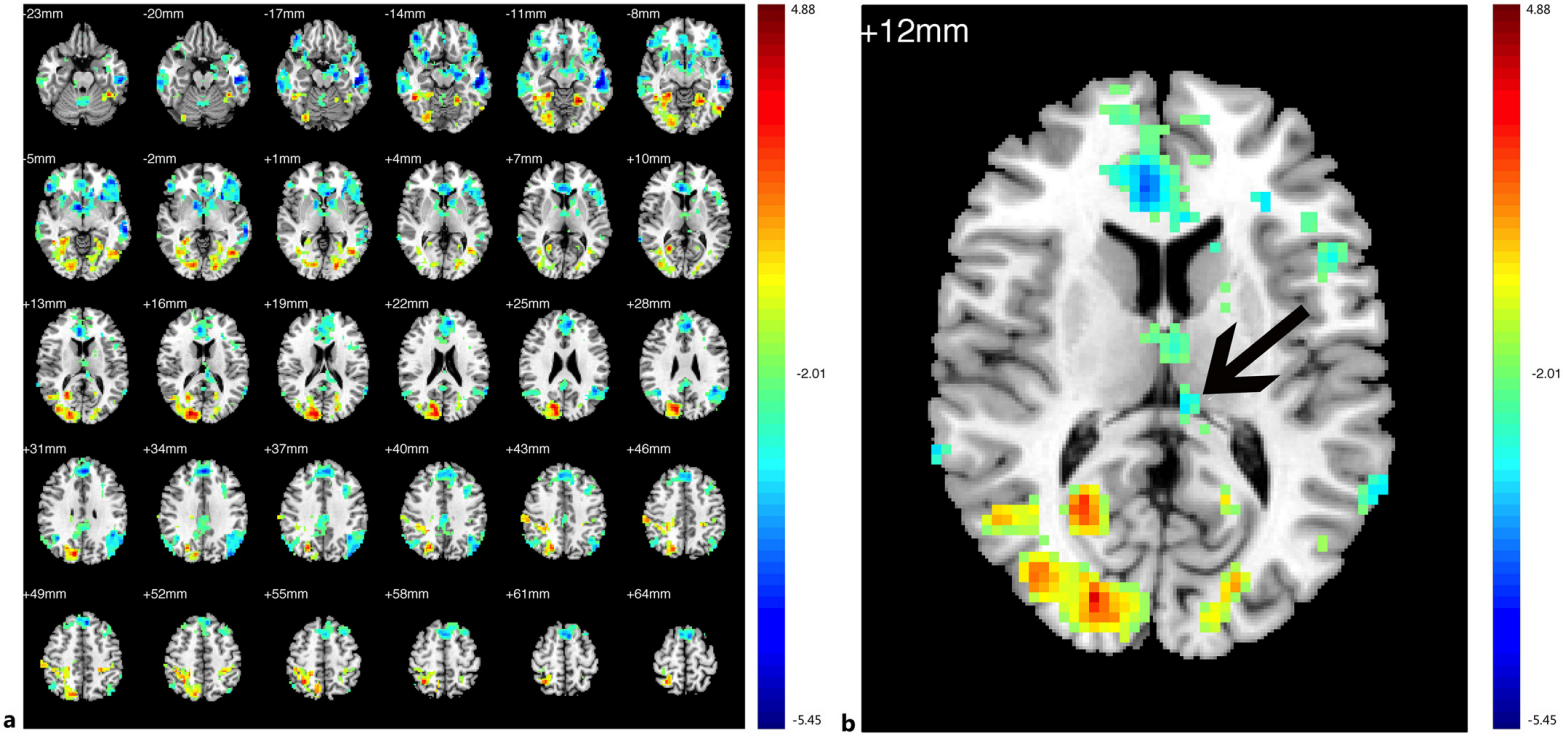
Brain areas exhibited significantly different FCs with the left V2 in PAs compared with HCs. (a) Statistical parametric map (axial view). (b) The dark arrow indicates left thalamus pulvinar. The cyan-blue colors indicate increased FC with the left V2 in the PAs versus the HCs, whereas the yellow-red colors indicate decreased FC for the same comparison (p<0.05, AlphaSim corrected).

**Fig 6:**
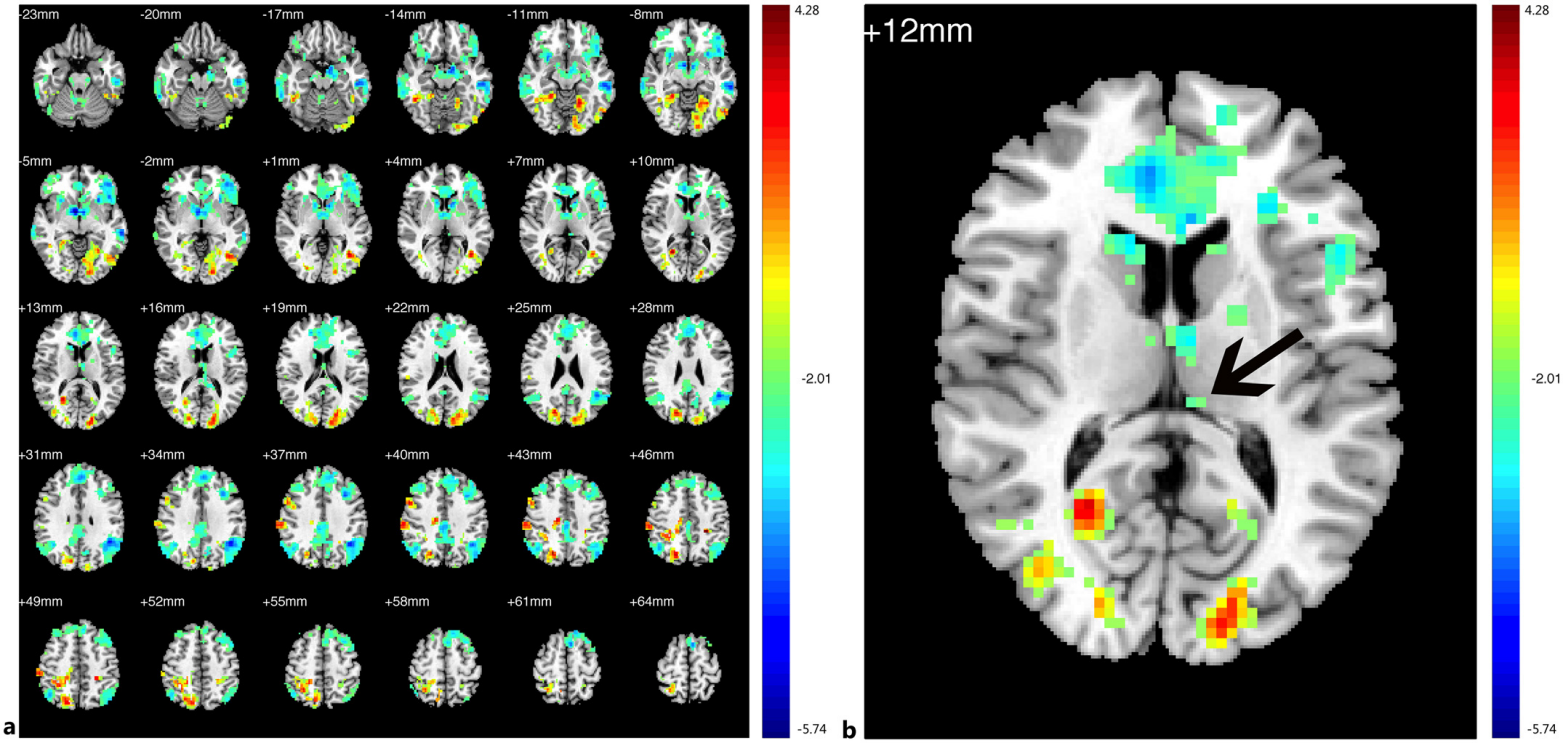
Brain areas exhibited significantly different FCs with the right V2 in PAs compared with HCs. (a) Statistical parametric map (axial view). (b) The arrow indicates left thalamus pulvinar. The cyan-blue colors indicate increased FC with the right V2 in the PAs versus the HCs, whereas the yellow-red colors indicate decreased FC. (p<0.05, AlphaSim corrected).

**Fig 7:**
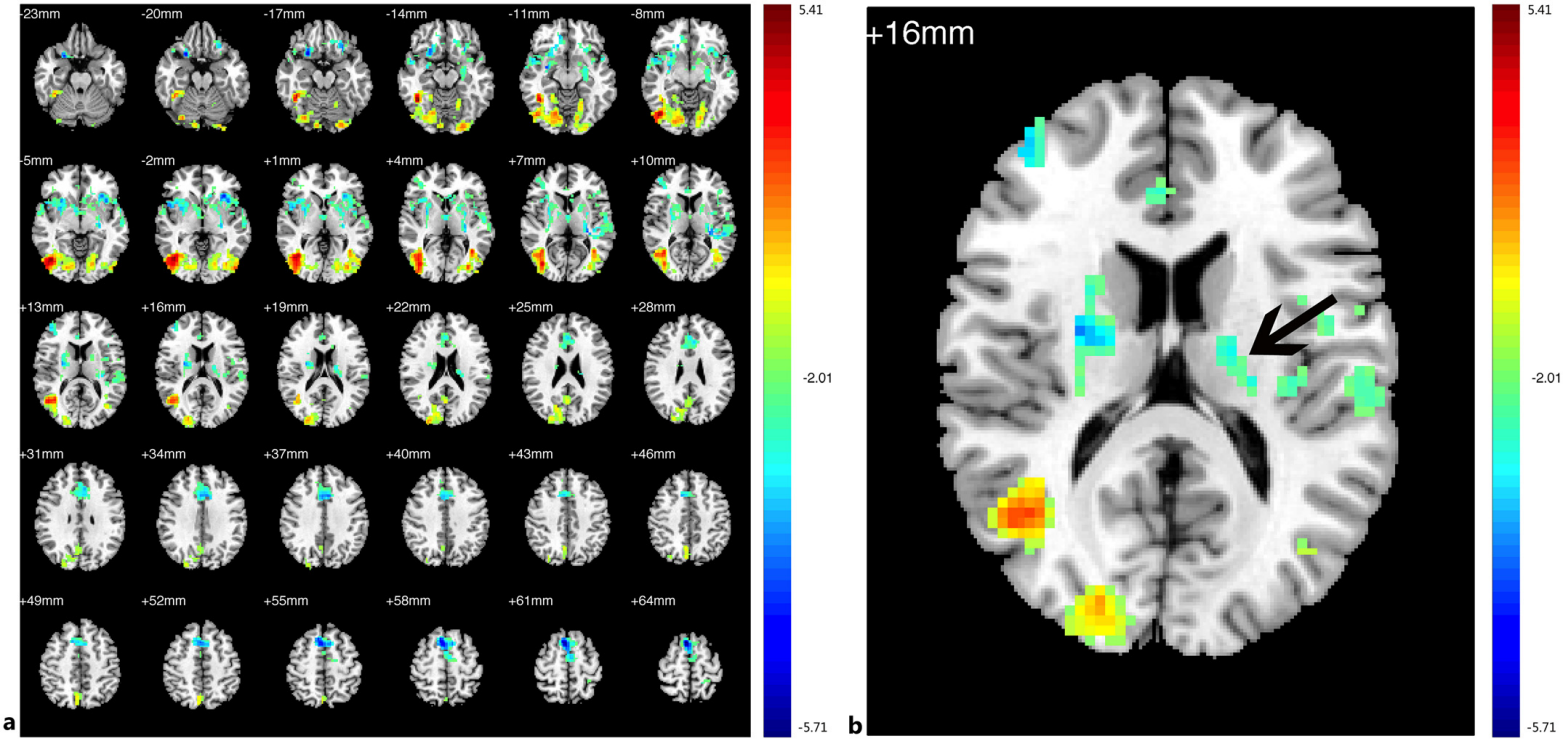
Brain areas exhibited significantly different FCs with the left MT+ in PAs compared with HCs. (a) Statistical parametric map (axial view). (b) The arrow indicates part of left thalamus. The cyan-blue colors indicate increased FC with the left MT+ in the PAs compared with the HCs, whereas the yellow-red colors indicate decreased FC. (p<0.05, AlphaSim corrected).

**Fig 8:**
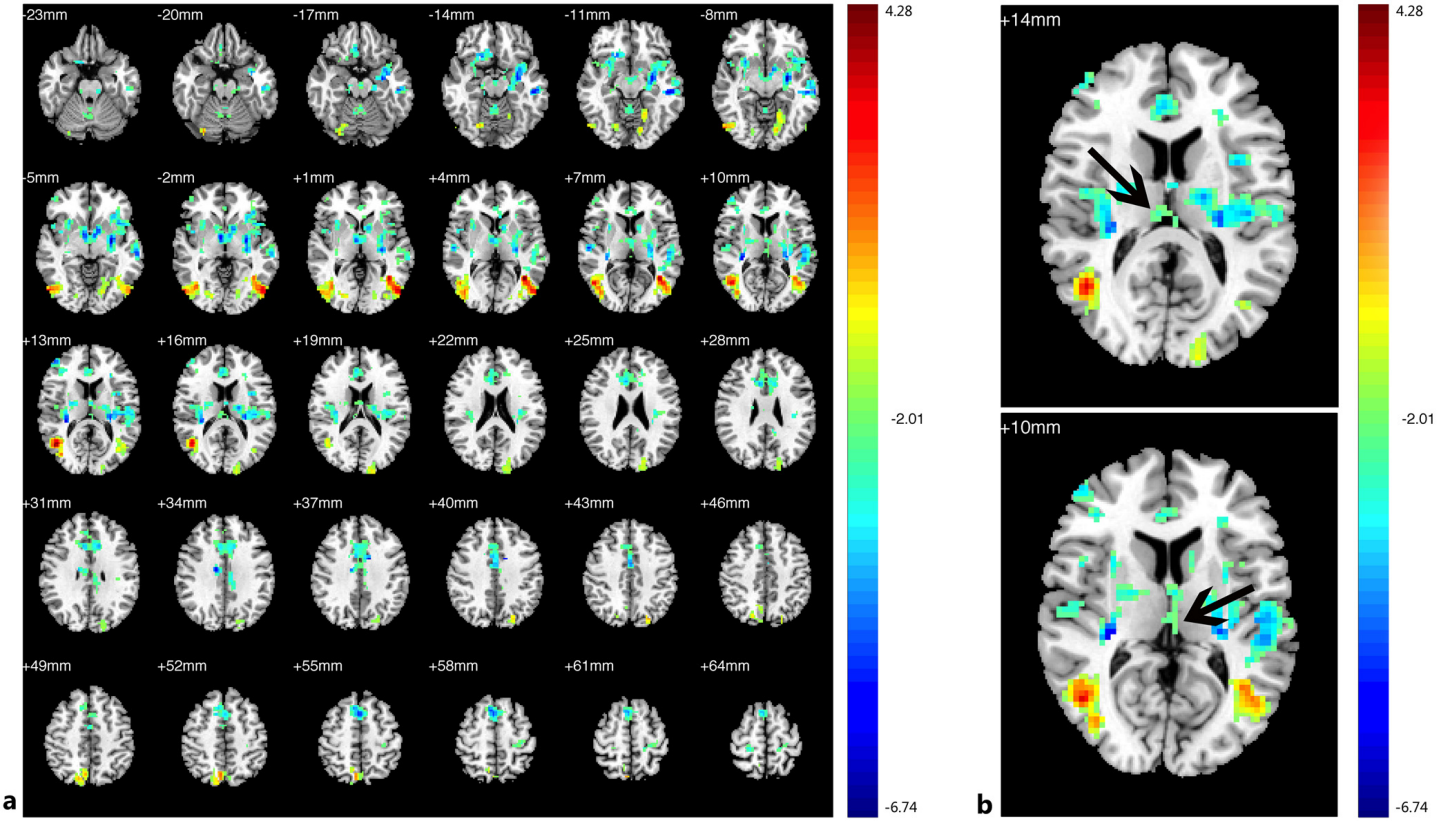
Brain areas exhibited significantly different FCs with the right MT+ in PAs compared with HCs. (a) Statistical parametric map (axial view). (b) The arrow indicates parts of thalamus. The cyan-blue colors indicate increased FC with the right MT+ in the PAs versus the HCs, whereas the yellow-red colors indicate decreased FC. (p<0.05, AlphaSim corrected).

Compared with the HCs, decreased FC in the PAs was identified between the left V1 and the bilateral V1, V2, right V3, and thalamus; increased FC with left V1 was identified in the bilateral middle and inferior temporal gyri (BA 20, 21), prefrontal cortex, ACC, angular gyrus, parahippocampal gyrus (PH), left SMG (Fig 3,Table 4, See the original data in S2 File)). Compared with the HCs, the PAs exhibited decreased FC between the right V1 and bilateral V1, V2, V3; increased FC was identified between the right V1 and the bilateral middle and inferior temporal gyri (BA 20 and 21, especially the left side), prefrontal cortex, PCC, ACC, insula, angular gyrus, bilateral hippocampus, PH, prefrontal cortex, left angular and left pre-SMA (Fig 4, Table 5, See the original data in S3 File). Compared with the HCs, the PAs had decreased FC between the left V2 and the bilateral V1, V2, V3, MT+, BA37, fusiform, and right SMG; increased FC was identified between the left V2 and the bilateral middle and inferior temporal gyri (BA 20, 21), ACC, prefrontal cortex, PCC, pre-SMA, angular gyrus, insula, caudate nucleus, and the left thalamic pulvinar (Fig 5, Table 6, See the original data in S4 File). Compared with the HCs, the PAs exhibited decreased FC between the right V2 and the left V1, V2, bilateral V3, MT+, BA37, right SMG, precentral gyrus, and fusiform; increased FC was identified between the right V2 and the bilateral middle and inferior temporal gyri (BA 20 and 21), ACC, prefrontal cortex, PCC, insular, pre-SMA, angular gyrus, bilateral caudate nucleus, and left thalamic pulvinar (Fig 6,Table 7, See the original data in S5 File). Compared with the HCs, the PAs had decreased FC between the left MT+ and the bilateral MT+, V1, V3, BA37, right V2, right fusiform, and bilateral precuneus cortex; increased FC was identified between the left MT+ and the bilateral insula, prefrontal cortex, pre-SMA, ACC, putamen, amygdaloid body, superior and middle temporal gyri (STG/MTG, BA20,21,22,41,42), and some regions of the thalamus (Fig 7,Table 8, See the original data in S6 File). Compared with the HCs, the PAs exhibited decreased FC between the right MT+ and the bilateral MT+, as well as some portions of the bilateral V2, and V3, BA37, precuneus; increased FC was identified between the right MT and the bilateral insula, pre-SMA, caudate nucleus, putamen, postcentral G, ACC, superior and middle temporal gyri, thalamus (especially the right thalamic pulvinar), left hippocampus (Fig 8, Table 9, See the original data in S7 File).

**Table 4:** Brain areas exhibited significantly different FCs with the left V1 in PAs compared with HCs.

Brain region	Peak intensity	Peak MNI coordinate			Cluster size (voxels)
		x	y	z	
**V1(L)**	2.48349	-1	-84	7	29*
**V1(R)**	2.70535	11	-81	3	29*
**V2(L)**	2.59074	-6	-74	17	53*
**V2(R)**	2.66061	13	-78	29	53*
**V3(L)**	2.92999	-16	-56	-14	17
**Thalamus(L)**	3.23376	-13	-18	0	40
**Thalamus(R)**	3.45907	16	-19	0	57
**ITG/MTG(L,BA20,21)**	-4.19997	-50	-25	-18	105
**ITG/MTG(R,BA20,21)**	-3.70392	30	-24	-18	161
**Prefrontal(L)**	-3.6293	-9	27	—9	99
**Prefrontal(R)**	-3.02146	8	42	-5	26
**ACC(L)**	-3.10297	-7	24	-7	55
**ACC(R)**	-3.33943	9	40	-4	59
**Angular Gyrus(L)**	-3.24624	-44	-50	25	44
**SMG(L)**	-3.30917	-9	10	69	16
**PHA(L)**	-3.34795	-12	-3	-18	17
**PHA(R)**	-3.70392	29	-24	-18	59

**Table 5:** Brain areas exhibited significantly different FCs with the right V1 in PAs compared with HCs.

Brain region	Peak intensity	Peak MNI coordinate			Cluster Size (voxels)
		x	y	z	
**V1(L)**	3.1239	-19	-54	5	6*
**V1(R)**	3.64646	21	-55	8	6*
**V2(L)**	2.11326	-19	-59	4	33*
**V2(R)**	2.29914	7	-84	11	33*
**V3(L)**	3.36925	-18	-56	-11	15*
**V3(R)**	3.95182	24	-55	9	15*
**ITG/MTG(L,BA20,21)**	-4.46491	-52	-23	-18	92
**ITG/MTG(R,BA20,21)**	-2.26346	61	-21	-18	32
**Prefrontal(L)**	-2.97517	-5	37	-14	101*
**Prefrontal(R)**	-3.34339	8	36	-11	101*
**ACC(L)**	-3.01843	-4	27	-6	58
**ACC(R)**	-3.11891	6	40	12	71
**PCC(L)**	-2.07709	-9	-49	27	38
**PCC(R)**	-2.27548	4	-36	29	16
**Angular G(L)**	-3.10513	-45	-53	29	86
**SMA(L)**	-2.64746	-7	18	66	60
**Hippocampus(L)**	-3.52553	-18	-12	-15	50
**Hippocampus(R)**	-2.32907	32	-27	-10	18
**PHA(L)**	-4.22966	-12	-2	-17	42
**PHA(R)**	-2.49999	31	-29	-15	64

**Table 6:** Brain areas exhibited significantly different FCs with the left V2 in PAs compared with HCs.

Brain region	Peak intensity	Peak MNI coordinate			Cluster size (voxels)
		x	y	z	
**V1(L)**	2.45636	-18	-84	8	5
**V1(R)**	2.19626	10	-83	-3	4
**V2(L)**	3.54017	-19	-81	-1	34
**V2(R)**	3.8735	10	-99	17	116
**V3(L)**	4.07261	-19	-55	-13	34
**V3(R)**	4.09363	13	-83	23	116
**MT+BA37(L)**	1.65922	-46	-62	-1	46
**MT+BA37(R)**	1.33574	43	-44	-16	28
**Fusiform(L)**	3.30535	-21	-46	-10	80
**Fusiform(R)**	3.93961	25	-43	-10	203
**Thalamus(L)**	-2.67332	-8	-26	13	22
**Prefrontal(L)**	-3.94889	-7	46	31	483*
**Prefrontal(R)**	-3.69608	3	46	31	483*
**PCC(L)**	-2.90701	-4	-38	32	34
**PCC(R)**	-2.4592	5	-44	24	36
**Insular(L)**	-3.52375	-35	17	-13	87
**Insula(R)**	-4.42491	29	18	-13	70
**Pre-SMA(L)**	-3.77456	-5	16	59	164
**Pre-SMA(R)**	-3.46695	3	15	59	74
**SMG(R)**	3.13667	59	-27	45	74
**ACC(L)**	-3.22473	-2	36	4	165
**ACC(R)**	-3.99956	5	39	12	201
**Caudate(L)**	-3.33452	-11	14	3	62
**Caudater(R)**	-2.94733	10	15	1	37
**Angular G(L)**	-4.4137	-48	-50	25	87
**Angular G(R)**	-3.29547	-47	-52	29	70
**MTG,ITG(L,BA20,21)**	-4.97988	-58	-22	-12	193
**MTG,ITG(L,BA20,21)**	-3.36614	60	-13	-12	96

**Table 7:** Brain areas exhibited significantly different FCs with the right V2 in PAs compared with HCs.

Brain region	Peak intensity	Peak MNI coordinate			Cluster size (voxels)
		x	y	z	
**V1(L)**	2.12035	-21	-59	12	9
**V2(L)**	3.32267	-14	-84	-4	129
**V3(L)**	3.25764	-29	-56	-6	128
**V3(R)**	3.60352	20	-78	26	13
**MT+BA37(L)**	3.42259	-45	-63	-2	30
**MT+BA37(R)**	1.31255	39	-62	6	37
**Fusiform(L)**	3.1962	-21	-48	-11	12
**Fusiform(R)**	3.45578	24	-41	-11	203
**Thalamus(L)**	-2.85544	-6	-23	14	27
**ACC(L)**	-2.78913	-6	39	5	194
**ACC(R)**	-3.66039	6	37	5	238
**PCC(L)**	-2.99933	0	-36	32	34
**PCC(R)**	-2.96533	4	-36	32	36
**Insular(L)**	-4.12405	-30	22	-8	88
**Insula(R)**	-3.71116	27	21	-11	48
**Pre-SMA(L)**	-3.72945	-4	16	58	164
**Pre-SMA(R)**	-2.16312	2	16	59	74
**SMG(R)**	3.44727	59	-23	40	105
**Precenrtal G(R)**	3.49703	51	5	39	75
**prefrontal(L)**	-3.95789	-2	48	30	622*
**prefrontal(R)**	-2.3762	7	53	33	622*
**Caudate(L)**	-4.35426	-12	19	2	62
**Caudater(R)**	-3.65652	10	16	2	89
**Angular G(L)**	-5.20743	-44	-50	31	307
**Angular G(R)**	-39095	41	-59	46	301
**MTG/ITG(L)**	-3.9697	-55	-22	-14	126
**MTG/ITG(R)**	-3.51063	65	-23	-18	72

**Table 8:** Brain areas exhibited significantly different FCs with the left MT+ in PAs compared with HCs.

Brain region	Peak intensity	Peak MNI coordinate			Cluster size (voxels)
		x	y	z	
**V1(L)**	2.05691	-10	-76	-4	9
**V2(L)**	3.5219	-26	-86	-17	78
**V2(R)**	3.23193	11	-87	-17	128
**V3(L)**	2.99225	-34	-84	1	56
**V3(R)**	5.12958	45	-70	-6	16
**Fusiform(R)**	5.02492	43	-46	-14	214
**Precuneus(L)**	2.10599	-3	-62	26	34
**Precuneus(R)**	2.79595	7	-66	47	116
**Thalamus(L)**	-2.55022	-19	-17	17	5
**ACC(L)**	-3.07972	-1	37	25	78
**ACC(R)**	-2.02499	3	39	15	79
**Postcentral G(L)**	-3.64972	-21	-39	75	51
**Insular(L)**	-3.77743	-32	25	-2	108
**Insula(R)**	-3.19933	46	11	-2	104
**Pre-SMA(L)**	-3.95592	-8	16	56	210
**Pre-SMA(R)**	-4.76096	7	18	59	216
**prefrontal(L)**	-4.02975	-27	31	-17	83*
**prefrontal(R)**	-3.51123	11	47	-12	83*
**Amygdaloid body(L)**	-2.54516	-25	-6	-14	16
**Amygdaloid body (R)**	-2.74615	29	1	-14	7
**Putamen (L)**	-2.39453	-25	3	-2	108
**Putamen (R)**	-2.63374	28	3	4	148
**MT+BA37(L)**	4.55961	42	-64	-2	60
**MT+BA37(R)**	3.05294	-48	-68	1	17
**STG/MTG(L) (BA20,21,22,41,42)**	-2.57516	-54	-29	8	173
**STG/MTG(R) (BA20,21,22,41,42)**	-2.98396	51	13	-11	98

**Table 9:** Brain areas exhibited significantly different FCs with the right MT+ in PAs compared with HCs.

Brain region	Peak intensity	Peak MNI coordinate			Cluster size (voxels)
		x	y	z	
**V2(L)**	3.06095	-15	-75	-10	46
**V2(R)**	2.10992	21	-75	-2	8
**V3(L)**	3.26141	-51	-77	-2	74
**V3(R)**	3.27105	52	-74	-2	44
**Precuneus(L)**	2.09516	-1	-73	54	12
**Precuneus(R)**	3.33504	7	-73	51	74
**Postcentral G(L)**	-3.2697	22	-34	68	58
**Postcentral G(R)**	-2.94379	-18	-34	71	41
**ACC(L)**	-2.31313	-3	40	2	98
**ACC(R)**	-2.36409	1	40	0	119
**Insular(L)**	-2.5325	-35	11	-11	169
**Insula(R)**	-2.56662	48	12	-2	57
**Pre-SMA(L)**	-3.26692	-1	13	59	91
**Pre-SMA(R)**	-3.31129	5	15	58	154
**Caudate nucleus(L)**	-3.13996	-9	12	2	40
**Caudate nucleus(R)**	-2.18396	9	14	-3	19
**Hippocampus (L)**	-4.35972	-29	-2	-11	41
**Putamen (L)**	-3.01153	-13	11	-5	117
**Putamen (R)**	-2.72684	30	16	-2	52
**MT+BA37(L)**	4.09914	-43	-59	4	22
**MT+BA37(R)**	4.75144	44	-59	13	26
**Thalamus(L)**	-2.06694	-3	-23	9	37
**Thalamus(R)**	-2.40108	6	-20	14	10
**STG/MTG (L,BA20,21,22,41,42)**	-4.17563	-53	-20	-15	132
**STG/MTG (R,BA20,21,22)**	-3.22972	55	-16	4	8

## Discussion

RS-fMRI is an emerging way to explore functional brain connectivity with minimal bias towards specific functions; it exhibits great advantage, particularly when subjects fail to perform active tasks [28]. Functional networks utilized by the active brain are continuously and dynamically “active” even “at rest”, which indicates the full repertoire of functional dynamics can be investigated with RS-fMRI [29].

The present study demonstrated that the ReHo of spontaneous brain activity and the connectivity patterns of vision-related networks remarkably change in PAs. Compared with the HCs, the PAs exhibited reduced ReHo or FC with ROI seeds primarily within the visual cortex (bilateral V1, V2, V3, fusiform, BA37, and MT+), whereas increased ReHo and FC were observed with ROI seeds primarily in the higher cognitive area (prefrontal cortex, PCC, insula, SMG, pre-SMA, angular gyrus, ACC, putamen, hippocampal formation, and caudate nucleus, BA20, 21, 22). It is worth mentioning that compared with HCs, V1 in PAs exhibited decreased or similar FC with the thalamus, whereas V2 and MT+ exhibited increased FCs with the thalamus, especially pulvinar.

### Functional changes in the earlier stages of the visual cortex and areas MT+/V5

ReHo reflects the local synchrony of neural activity [35]. FC coordinates and integrates activity throughout anatomically distributed but functionally related neural areas across space and time [26]. Increased ReHo or FC may reveal an abnormal enhancement of intraregional/interregional neural activity [26,35]. In contrast, decreased ReHo or FC reflects degradation of the synchronization between intraregional/interregional neural activity and implies functional deficits [26,35]. In our study, the thalamus, an important relay nucleus for conscious visual information, and most subareas within the visual cortex (V1, V2, V3, MT+, BA37, and fusiform) exhibited significantly decreased ReHo in the PAs, which suggests neural dysfunction in these areas. The decreased FCs between most subareas within the visual cortex and the 6 ROIs also implies neural disconnection within the visual cortex that is associated with anterior visual pathway disease. In terms of the FC with the thalamus, V1 exhibited no significant difference between the two groups or significant decreases in PAs; however, MT/V5+ and V2 exhibited significant increases in the PAs. Many studies have identified preserved visual function despite a damaged primary visual cortex, especially in the immature visual cortex [10, 11, 36]. However, few studies (only 2 studies to our knowledge) have recruited patients with anterior vision pathway disease rather than a V1 lesion [37, 38]. Giulia Mascioli used visual stimulation fMRI to study the visual cortex of optic neuritis patients and found that compared with the unaffected eye, the affected eye exhibits a significantly lower BOLD signal in area V1, whereas no significant difference was identified in area V5. These results indicate that the thalamus—V5 bypass is less affected by optic neuritis, or perhaps this pathway plays a compensatory role for the vision loss observed in anterior vision pathway diseases [37]. Compared with normal controls, Giulia Dormal identified an increased response to visual motion information in the bilateral V5 in one low-vision patient (female, age 41); this study also used vision-task fMRI and reported reduced responses to visual motion information after visual restoration compared with prior to operation, which augmented the compensation of V5/MT+ for poor vision and compensation attenuation after vision restoration [38]. Consistent with our study, both of these results suggest that V5/MT+ potentially compensate for the decrease in visual information from V1, even if the total amount of visual information from the thalamus has decreased. Thus, the bypass from the thalamic pulvinar or LGN to V5/MT+ plays an important role in the reorganization of the visual cortex and the recovery of vision loss in anterior visual pathway diseases.

In our study, we also identified increased FC between the V2 (especially the right side) and the thalamic pulvinar in the PAs compared with the HCs. Unlike the LGN, which is the major relay to the cortex from the retina, the pulvinar relays only minor retinal input to the visual cortex, often in PIm, and provides a very direct extrageniculate pathway to the MT [39, 40]. Substantial input to the pulvinar originates from the visual cortex, and a substantial projection from the pulvinar terminates in the visual cortex, including V2. This strong two-way connection is likely modulatory [40]. It has been noted that the projections to V2 from the pulvinar are largely from retinotopically matched locations in the PLvl and PIcl. In addition, the PL powerfully controls and gates the information outflow from the V1 to extrastriate areas [41]. Soares et al., working in the Cebus monkey, identified a mixture of both excitation and inhibition of V2 responses after pulvinar blockade with gamma-aminobutyric acid [42]. Thus, it appears the PL is situated to not only directly activate V2 but also to control the V1 signals that influence V2. Taken together, these two studies emphasize a critical role for thalamocortical connections in determining the propagation of sensory information throughout the cortex [43]. It is easy to imagine a scenario in which the signal from V1 to V2 decreases because of anterior visual pathway disease, whereas the information feedback to the pulvinar enhances the modulation of V2 from the pulvinar most likely as a compensatory mechanism. This hypothesis is consistent with our results from PAPVDs, which indicate decreased visual information from V1 to V2 associates with increased FC between the V2 and pulvinar.

### Functional changes in the higher cognitive cortex within DMN and SN

In our ReHo analysis, we identified significantly increased ReHo values within the bilateral precuneus gyrus, prefrontal cortex, ACC, PCC, insula, SMG, and putamen in the PAs compared with the HCs. In the FC analysis, we identified increased FC with ROIs in the bilateral temporal lobe (especially BA20,21,22), prefrontal cortex, PCC, insular, angular gyrus, ACC, pre-SMA, SMG, hippocampal formation, caudate and putamen in the PAs compared with the HCs. All of these areas, with the exception of the inferior temporal gyrus, caudate nucleus, and putamen, are subareas of either the DMN or SN.

Nowadays more and more studies have demonstrated that various etiologies could cause functional reorganization in DMN and SN. A significantly increased and reduced integration of DMN areas in the hippocampal and prefrontal regions, respectively, were demonstrated in glioma patients. Modifications were closely related to tumor grading [43,44] Diffuse gliomas in the left parietal lobe showed a more impaired DMN compared with tumors in the frontal lobe, while tumors within and outside the network nodes did not differ significantly[45]. Increased DMN FC correlates with improved cognitive outcome after resective surgery in glioma patients [46]. Functional changes within the DMN and SN were confirmed in patients with HBV-related cirrhosis [47], Parkinson’s disease, depression and other cognition-impaired patients. [48]. Increased ReHo in the right anterior insular cortex, a key node in SN, as well as in the left parahippocampal cortex (lPHC), a key node in the DMN in unilateral hearing damage patients were confirmed. Moreover, seed-based resting–state functional connectivity analysis showed an enhanced relationship between rAI, a key node in the SN, and several key regions of the DMN [49].

However, the mechanism resulting in functional alteration in DMN or SN is still to be elucidated. It is known that the DMN exhibits task-induced deactivation during various cognitive tasks [50–57]. Stephen D. Mayhew demonstrated that a nonlinear reduction in visual PBR and DMN NBR occurred in visual task trials that were preceded by high alpha-power [58]. Decreased pre-stimulus cortical excitability (higher alpha-power) is associated with subsequent stimuli that are less engaging and evoke smaller amplitudes of visual PBR, which results in a milder interruption of intrinsic processes and decreased DMN deactivation (increased BOLD level) [58–61]. Jue Mounder also demonstrated that strong DMN activity is associated with reduced visual cortical excitability in healthy people [62]. Similarly, our study determined that lower ReHo values in V1 associated with higher ReHo in the DMN along with enhanced FC between V1 and the DMN. Given that the DMN is involved in the detection and monitoring of both environmental events and internal mentation [57] and is posited to modulate subject responsiveness (cortical excitability) and the saliency of external stimuli [51, 52,57,63], the detection of decreased visual cortical activity by the DMN is likely to incur the decreased deactivation in DMN. So it may be justified to propose that decreased visual cortex activity in some way plays a role in decreased DMN deactivation (stronger activity). The SN, composed of the presupplementary motor (pre-SMA)area and anterior insula,[55, 64, 65], facilitates task-related information processing and magnify specific external stimuli by deactivating the DMN [50,55,66–69]. Because the SN plays an important role in detection, adjusting and biasing sensory stimuli for top-down attentional control [70, 71], enhanced functional connectivity between the visual system and the SN is justified if visual stimulation is decreased. The anatomical connection between the visual cortex and the SN has been demonstrated [72]. Taken together, it is reasonable to posit that when visual cortex dysfunction is detected, higher than normal SN activity is initiated to achieve the saliency mechanism, as well as to deactivate the correspondingly increased DMN activity in the brain network.

Similarly, maybe we could surmise that decreased activity of brain cortex in charge of environmental events sensation due to various diseases may be likely to underlie in functional changes in DMN and SN in various etiology. Definitely, more studies are necessary to be carried out to confirm it.

In addition, Stein, M.B. et al. demonstrated that AI hyperactivity is implicated in anxiety disorders [73]. Because patients are often more anxious than controls, the association between anxiety and AI hyperactivity cannot be excluded in our study.

In our study, the precuneus demonstrated complicated functional changes. Compared with the HCs, ReHo increased and FC exhibited no significant differences with the left V1, bilateral V2, or right MT+, whereas there was a decrease in the left MT+ and an increase in the right V1. In their study, Daniel et al. demonstrated that the precuneus is composed of three discrete subdivisions: (i) the anterior precuneus, which specializes in sensorimotor processing, (ii) the central precuneus, which specializes in cognition, and (iii) the posterior precuneus, which specializes in vision [74]. It is likely that this complexity results from the multiple functions and heterogeneity of this area.

We are aware that the present study might have the following limitations: 1) we attributed the enhancement of FC between MT+ and V2 to the compensatory mechanism of the pulvinar; however, we did not define the pulvinar in the thalamus with exact ordinates. 2) The definition of MT+ was based on previous studies instead of task fMRI definition, which may lead to some bias. 3) Because pituitary tumors are located in the sellar region, the presence of direct compression to the hippocampus in some patients cannot be ruled out as an influence on our results. 4) Further studies are required to investigate the mechanism of the interactions between the DMN, SN and the visual network. 5) Visual acuity assessment was performed with the E chart without using more appropriate universal charts.

## Conclusion

In conclusion, visual impairment due to anterior visual pathway lesion can incur reconstruction within the visual cortex via the initiation of compensatory mechanisms in specific visual areas (MT+, V2). The functional changes in subareas within DMN and SN in PAs suggest that vision damage may precipitate to function remodeling in higher cognitive cortex beyond the visual cortex. However, additional studies are needed to unravel the mechanism of neural plasticity within the visual cortex, as well as the mechanism of the interaction between the visual and higher cognitive cortices.

## Supporting Information

S1 File The original data of the ReHo analysis between two groups. (NII)

S2 File The original data of the FC analysis with left V1 between two groups. (NII)

S3 File The original data of the FC analysis with right V1 between two groups. (NII)

S4 File The original data of the FC analysis with left V2 between two groups. (NII)

S5 File The original data of the FC analysis with right V2 between two groups. (NII)

S6 File The original data of the FC analysis with left MT between two groups. (NII)

S7 File The original data of the FC analysis with right MT between two groups. (NII)
